# Gastrokine 1 induces senescence and apoptosis through regulating telomere length in gastric cancer

**DOI:** 10.18632/oncotarget.2586

**Published:** 2014-12-10

**Authors:** Jung Hwan Yoon, Ho Seok Seo, Won Seok Choi, Olga Kim, Suk Woo Nam, Jung Young Lee, Won Sang Park

**Affiliations:** ^1^ Department of Pathology, College of Medicine, The Catholic University of Korea, Banpo-dong, Seocho-gu, Seoul 137-701, South Korea; ^2^ Department of General Surgery, College of Medicine, The Catholic University of Korea, Banpo-dong, Seocho-gu, Seoul 137-701, South Korea; ^3^ Functional RNomics Research Center, College of Medicine, The Catholic University of Korea, Banpo-dong, Seocho-gu, Seoul 137-701, South Korea

**Keywords:** GKN1, telomeres, telomerase, senescence, apoptosis

## Abstract

The present study aims to investigate whether gastrokine 1 (GKN1) induces senescence and apoptosis in gastric cancer cells by regulating telomere length and telomerase activity. Telomere length, telomerase activity, and hTERT expression decreased significantly in AGS^GKN1^ and MKN1^GKN1^ cells. Both stable cell lines showed increased expression of TRF1 and reduced expression of the hTERT and c-myc proteins. In addition, TRF1 induced a considerable decrease in cell growth, telomerase activity, and expression of hTERT mRNA and protein. GKN1 completely counteracted the effects of c-myc on cell growth, telomere length, and telomerase activity. Interestingly, GKN1 directly bound to c-myc and down-regulated its expression as well as inhibited its binding to the TRF1 protein and a *hTERT* promoter. Furthermore, GKN1 triggered senescence, followed by apoptosis via up-regulating the p53, p21, p27, and p16 proteins and down-regulating Skp2. Telomere length in 35 gastric cancers was shortened significantly compared with the corresponding gastric mucosae, whereas GKN1 expression was inversely correlated with telomere length and *c-myc* and *hTERT* mRNA expression. Taken together, these results suggest that GKN1 may shorten telomeres by acting as a potential c-myc inhibitor that eventually leads to senescence and apoptosis in gastric cancer cells.

## INTRODUCTION

Telomeres, which consist of a repetitive G-rich DNA sequence and telomeric protein, play an important role in the prevention of end-to-end chromosome fusion and genomic instability [1, 2, 3]. Telomeric chromatin is characterized by the association of telomeric DNA with specialized proteins that organize the linear chromosome end into a stable structure that is not recognized by the cell as a chromosome break [4]. Telomere length in healthy cells is highly regulated in a tissue- and cell type-specific manner and is dependent on mitotic turnover rate, telomerase activity, and telomerase-associated factors [5]. Several studies, including those in humans, have observed shortening of telomeres in vivo during aging [6]. Telomere shortening and resulting telomere dysfunction have been suggested to contribute to cancer susceptibility by increasing the risk of chromosomal aberrations caused by the breakage-fusion-bridge cycle [7]. Defects in telomere maintenance contribute to the initiation of genomic instability during carcinogenesis, including gastric cancer [8, 9]. Telomere maintenance in cancer cells is often accompanied by activated telomerase to protect genetically damaged DNA from normal cell senescence or apoptosis [10]. However, little is known about telomere maintenance in gastric mucosal epithelial cells and its contribution to gastric carcinogenesis.

Recently, Toback *et al*. reported that gastrokine 1 (GKN1) protects the antral mucosa and promotes healing by facilitating restoration and proliferation after injury [11]. In addition, GKN1 protects the intestinal mucosal barrier by acting on specific tight junction proteins and stabilizing peri-junctional actin [12]. We found previously that loss of GKN1 expression occurs frequently in gastric cancers and that GKN1 inhibits cell proliferation and induces apoptosis [13, 14]. Recently, it has been reported that GKN1 induces senescence by activating p16/Rb pathway in gastric cancer cells [15]. As replicative senescence is closely linked to shortening of telomeres and the p16/Rb pathway [16], we hypothesized that GKN1 may induce senescence of gastric cancer cells by regulating telomere length.

In this study, we focused on the effect of GKN1 on telomere maintenance and senescence in gastric cancer tissues and cell lines. Overall, we showed that GKN1 promotes senescence and apoptosis by regulating the length of telomeres in gastric cancers.

## RESULTS

### GKN1 suppresses cell growth and shortens telomeres in gastric cancer

**Figure 1:**
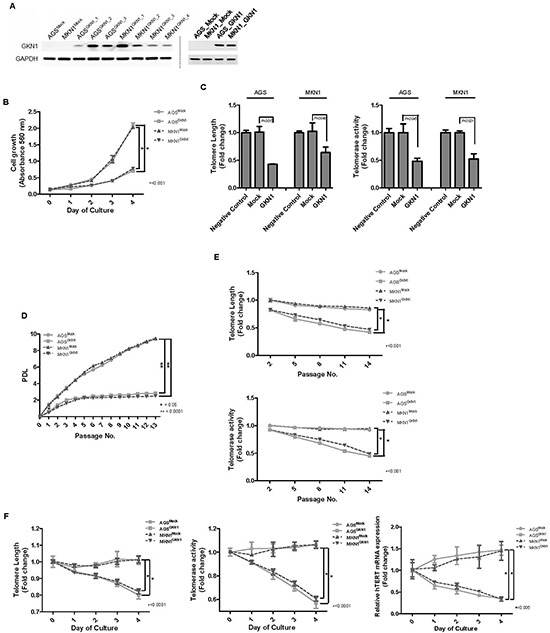
GKN1 induces telomeres shortening in gastric cancer cells **(A)** Stable GKN1 transfectants, AGS^GKN1^ and MKN1^GKN1^ (left panel), and transient GKN1 transfectants (right panel), showed marked expression of GKN1 by Western blot analysis. **(B)** In the MTT assay, GKN1 stable cells showed time-dependent inhibition of cell viability. **(C)** Telomere length and telomerase activity decreased significantly in AGS and MKN1 cells transiently transfected with *GKN1,* compared with those in non-transfected negative control and mock-transfected cells. **(D)** AGS^Mock^, MKN1^Mock^, AGS^GKN1^, and MKN1^GKN1^ cells were serially cultivated for 13 passages until the cells reached the end of their lifespan. The cumulative PDL diminished significantly after passage 1 and exhibited minimal proliferative activity after passage 4 in GKN1 stable transfectants. **(E)** A steady decline in telomere length and telomerase activity was observed with advancing passages in AGS^GKN1^ and MKN1^GKN1^ cells. **(F)** Stable transfectants from passage 5 showed significant shortening of telomeres, as well as decreased telomerase activity and *hTERT* mRNA expression in a time-dependent manner.

We stably reconstituted GKN1 in AGS and MKN1 cells. Stable transfectants of AGS^GKN1^ and MKN1^GKN1^ cells markedly expressed GKN1, as detected by Western blotting (Figure 1A). In MTT assays, AGS^GKN1^ and MKN1^GKN1^ stable cells grew at much slower rates than that of AGS^Mock^ and MKN1^Mock^ cells in a time dependent manner (Figure 1B). We tested telomere length in AGS and MKN1 cells after transient transfection with mock or *GKN1* to investigate whether the inhibition of cell growth by GKN1 is associated with the telomere attrition. Average telomere length and telomerase activity decreased significantly in *GKN1*-transfected AGS and MKN1 cells, compared to those in mock-transfected cells (Figure 1C). In addition, the cumulative population doubling levels (PDLs) decreased significantly after passage 1 (P = 0.0002 and P < 0.0001, respectively), and AGS^GKN1^ and MKN1^GKN1^ cells exhibited minimal proliferative activity after passage 4 (Figure 1D). Furthermore, unlike mock-transfected cells, AGS^GKN1^ and MKN1^GKN1^ cells that stably expressed GKN1 demonstrated shortened telomeres and diminished telomerase activity with additional passages (Figure 1E). These stable transfectants from passage 5 showed significant shortening of telomeres, as well as decreased telomerase activity and *hTERT* mRNA expression in a time-dependent manner (Figure 1F).

### GKN1 regulates the expression of telomere-related proteins

**Figure 2:**
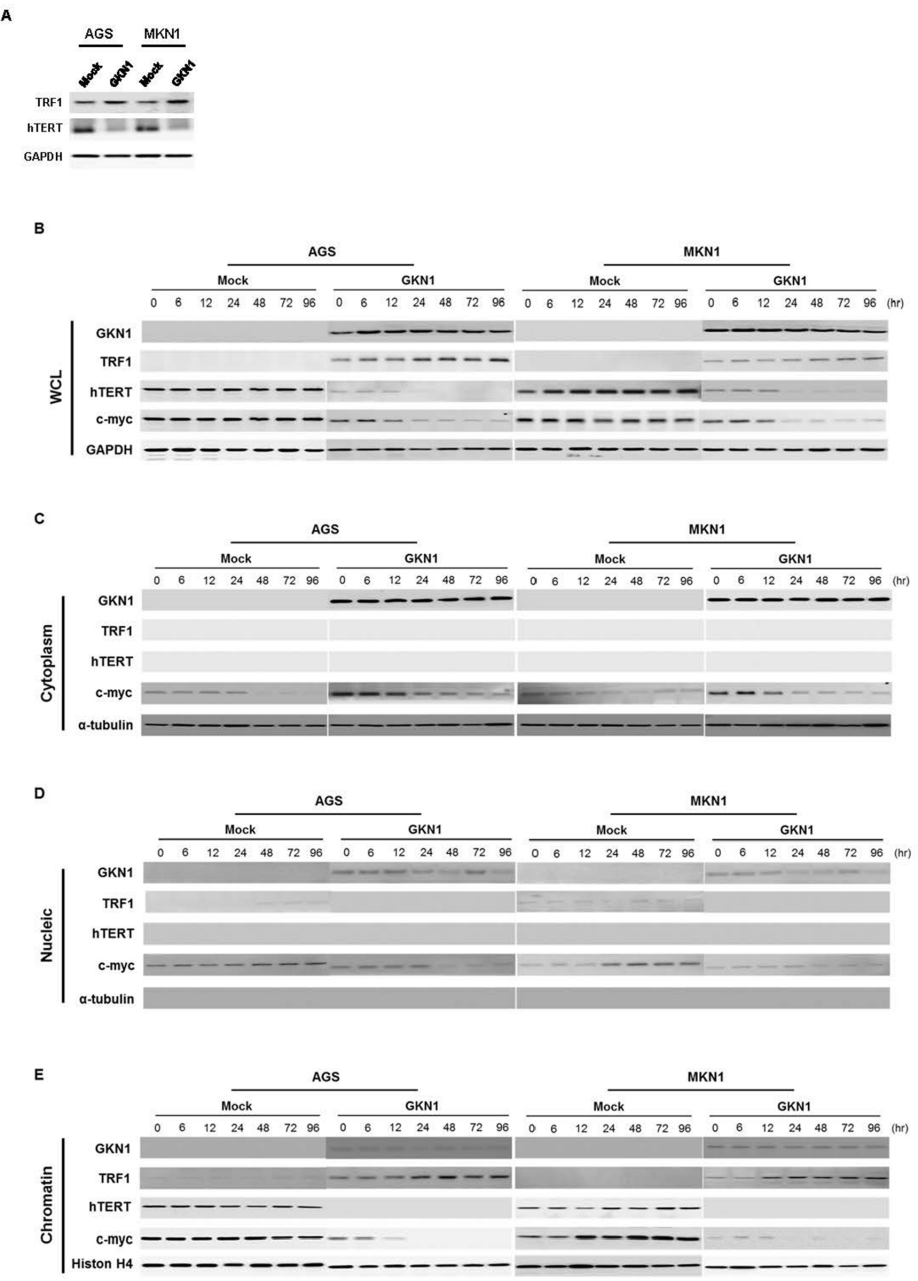
GKN1 negatively regulates expression of telomere-related proteins **(A)** AGS and MKN1 cells transiently transfected with GKN1 showed increased TRF1 and decreased hTERT expression levels. **(B)** GKN1 stable transfectants, AGS^GKN1^ and MKN1^GKN1^, induced TRF1 expression and down-regulated hTERT and c-myc protein expression in whole cell lysates (WCL). **(C)** In the cytoplasmic fraction of stable transfectants, GKN1 down-regulated c-myc expression. **(D)** GKN1 completely reduced TRF1 and c-myc protein expression detected in the nucleus of mock stable cells. **(E)** Increased expression of TRF1 and decreased expression of the c-myc and hTERT proteins were observed in the chromatin fraction of GKN1 stable transfectants. The accuracy of fractionation was confirmed with a cytoplasmic marker (α-tubulin) and a chromatin marker (histone H4).

To further validate whether shortened telomeres and reduced telomerase activity are dependent on GKN1, expression levels of the telomere-regulators, including TRF1, hTERT and c-myc, were examined in AGS and MKN1 cells. Both cell lines transiently transfected with *GKN1* showed up-regulation of TRF1 protein and down-regulation of hTERT (Figure 2A). In addition, we revealed increased TRF1 expression and reduced hTERT and c-myc protein expression in AGS^GKN1^ and MKN1^GKN1^ stable cells, compared to that in mock control cell lines (Figure 2B). Furthermore, we analyzed the localization of the TRF1, hTERT, and c-myc proteins after protein fractionation and found that c-myc protein expression was reduced in cytoplasm, nucleus, and chromatin of AGS^GKN1^ and MKN1^GKN1^ cells (Figure 2B-E). TRF1 expression in chromatin of GKN1 stable cells increased dramatically, whereas c-myc and hTERT expression was markedly abolished (Figure 2E).

Next, we examined the effect of TRF1 on cell growth, telomere length, and telomerase activity in AGS and MKN1 cells transfected with *TRF1*. In MTT assay, the ectopic expression of TRF1 resulted in a significant decrease in cell growth, compared to that in the mock-transfected cells (Supplementary Figure 1A). Additionally, time-dependent shortening of telomeres, decreased telomerase activity, and reduced hTERT mRNA and protein expression were observed in cells transfected with *TRF1* (Supplementary Figure 1B-E).

### GKN1 induces cellular senescence and apoptosis in gastric cancer cells

**Figure 3:**
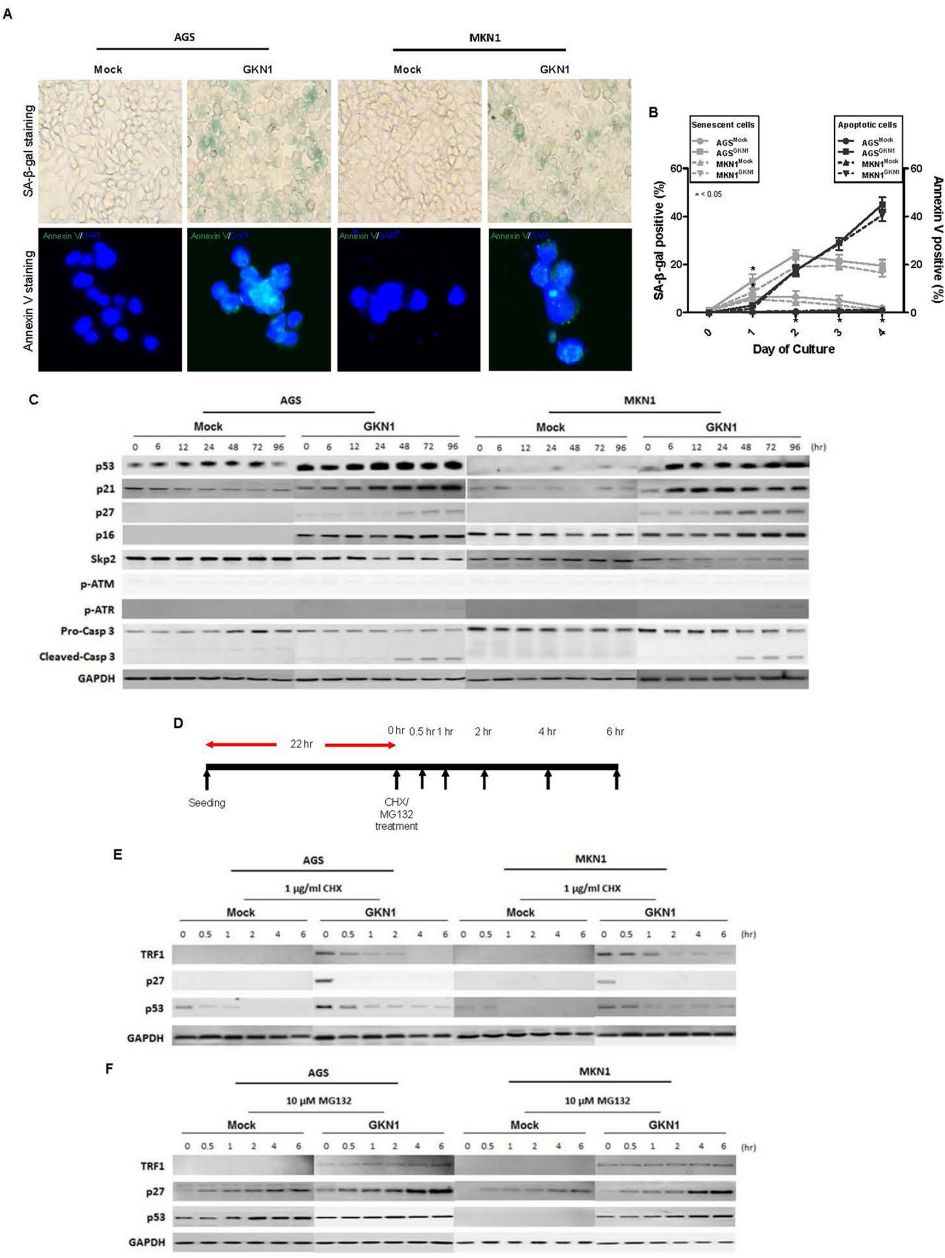
GKN1 induces cellular senescence and apoptosis **(A)** GKN1 or mock stable transfectants from passage 5 showed SA-β-gal- and Annexin V-positive staining at 48 hr. **(B)** The percentage of SA-β-gal-positive cells in AGS^GKN1^ and MKN1^GKN1^cells increased significantly by 24% and 19% at 48 hr and then decreased by 19.5% and 16.5% at 96 hr, respectively (*P < 0.05). In contrast, the percentage of apoptotic cell death detected by Annexin V staining increased in a time dependent manner, and a significant difference was detected after 48 hr (P < 0.05). **(C)** GKN1-reconstituted cells dramatically up-regulated the expression of p53, p21, p27, and p16 proteins and down-regulated expression of the Skp2 protein. In addition, cleaved caspase-3 expression was persistently detected after 48 hr in GKN1-reconstituted cells. **(D, E, F)**. AGS and MKN1 cells from passage 5 were exposed to CHX and MG-132 for 0, 0.5, 1, 2, 4 and 6 hr and total proteins were isolated (D). Cycloheximide abolished GKN1-induced TRF1, p53, and p27 expression (E), whereas MG-132 rescued degradation of these proteins in both control and GKN1 stable cells (F).

To detect cellular senescence, AGS^Mock^, MKN1^Mock^, AGS^GKN1^ and MKN1^GKN1^ cells from passage 5 were stained for SA-β-gal activity (Figure 3A). The mean percentage of SA-β-gal-positive AGS^GKN1^ and MKN1^GKN1^ stable cells increased by 13% and 5.5% at 24 hr and by 24% and 19% 48 hr (P = 0.0134 and P = 0.0247), respectively (Figure 3B). Interestingly, AGS^GKN1^ and MKN1^GKN1^ cells demonstrated a decrease in SA-β-gal activity at 72 hr (21.5% and 19.5%) and 96 hr (19.5% and 16.5%), whereas the percentage of apoptotic cell death increased significantly in a time-dependent manner, compared to those of mock control cells (Figure 3B). To further investigate the role of GKN1 in the induction of cellular senescence and apoptosis, we analyzed the expression of representative regulators, including p53, p21, p27, p16, Skp2, and caspase-3 proteins. We showed that GKN1-reconstituted cells were accompanied by pronounced up-regulation of the p53, p21, p27, and p16 proteins and reduced expression of the Skp2 protein. However, GKN1 did not affect the expression of p-ATM and p-ATR (Figure 3C). Consistent with the results of Annexin V staining, the cleaved form of caspase-3 was expressed steadily after 48 hr in GKN1-reconstituted cells (Figure 3C).

We subsequently examined the stability of the above proteins at 0, 0.5, 1, 2, 4, and 6 hrs after treatment with cycloheximide (CHX) and MG-132 in AGS^GKN1^ and MKN1^GKN1^ cells from passage 5. As shown in Figure 3D-E, TRF1, p53, and p27 protein expression levels induced by GKN1 decreased markedly in the presence of CHX, whereas MG-132 rescued degradation of these proteins in both mock control and GKN1 stable cells (Figure 3F), indicating that the TRF1, p53, and p27 proteins induced by GKN1 may be degraded by proteasomes.

### GKN1 regulates telomere length by targeting c-myc in gastric cancer cells

**Figure 4:**
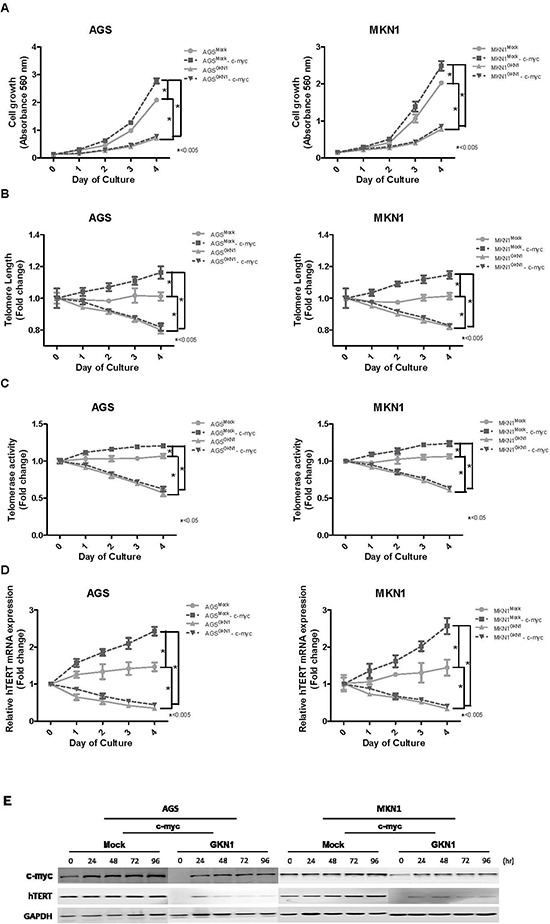
GKN1 regulates telomere length by targeting c-myc **(A)** AGS^Mock^, MKN1^Mock^, AGS^GKN1^ and MKN1^GKN1^ cells from passage 5 were transiently transfected with *c-myc.* A significant time-dependent increase in cell growth was observed in *c-myc*-transfected cells, whereas GKN1 completely counteracted c-myc-induced cell growth. **(B)** Ectopic expression of c-myc induced telomere elongation, but GKN1 significantly inhibited the effect of c-myc on telomere length in both cells. (C) c-myc slightly increased telomerase activity, whereas GKN1 markedly down-regulated telomerase activity in both cell types. **(D and E)** c-myc significantly up-regulated hTERT mRNA and protein expression levels in AGS^Mock^ and MKN1^Mock^ cells, whereas GKN1 suppressed c-myc-induced hTERT expression.

As shown in Figure 4A, a significant time-dependent enhancement in growth rates was observed in *c-myc*-transfected AGS and MKN1 cells, whereas GKN1 completely counteracted c-myc-induced cell growth. Ectopic expression of c-myc increased telomere length and telomerase activity in AGS^Mock^ and MKN1^Mock^ cells (Figure 4B and 4C). In contrast, c-myc did not affect telomere length or telomerase activity in either AGS^GKN1^ or MKN1^GKN1^ cells (Figure 4B and 4C). Furthermore, c-myc dramatically up-regulated *hTERT* mRNA expression in mock stable cells, but GKN1 significantly down-regulated *hTERT* mRNA expression in AGS^GKN1^ and MKN1^GKN1^ cells (Figure 4D). This result was also confirmed at the protein level by Western blot (Figure 4E). We repeated the experiments twice and found consistent data.

### GKN1 inhibits binding activity of c-myc to a hTERT and TRF1 promoter

**Figure 5:**
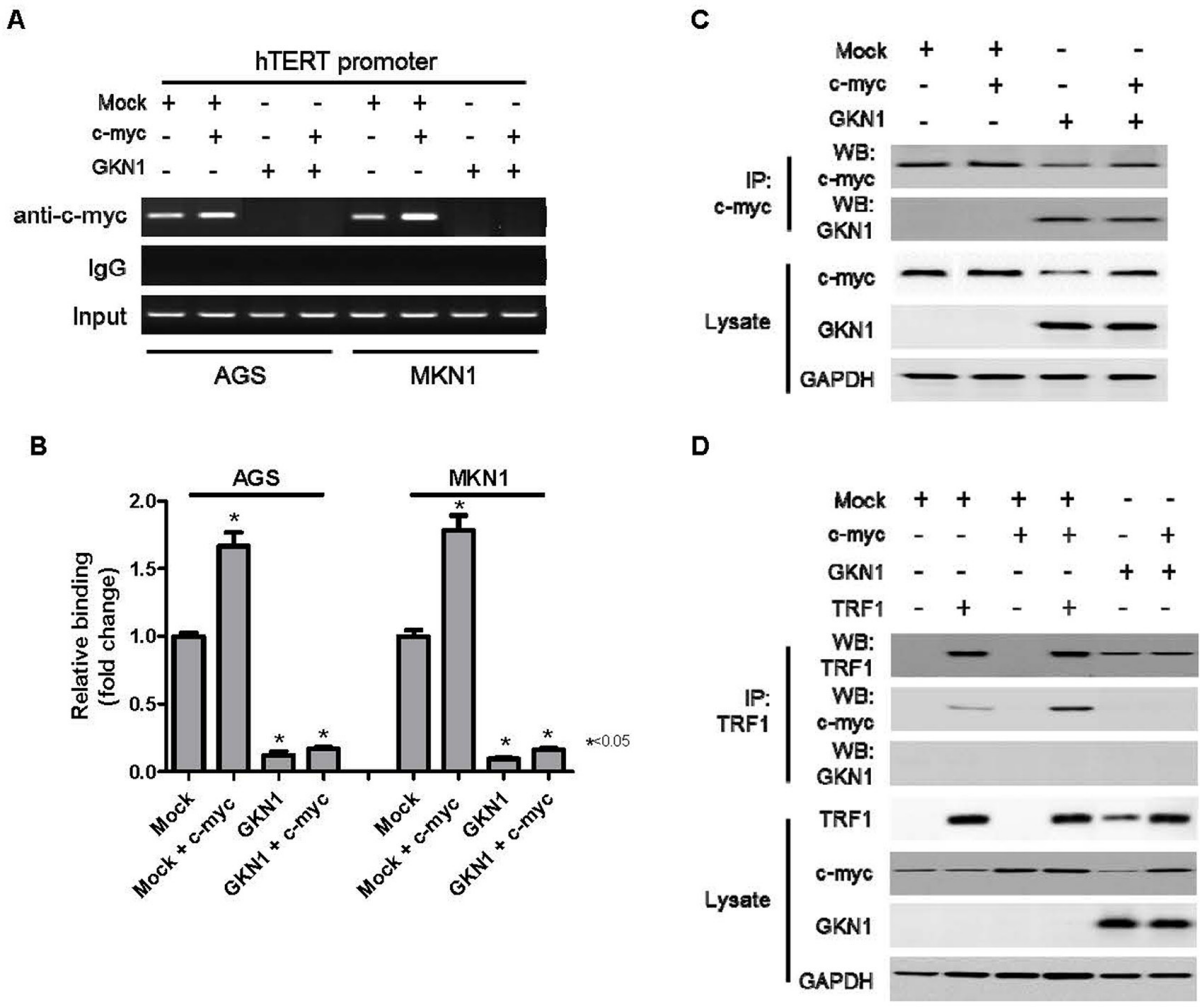
GKN1 directly binds to c-myc and inhibits c-myc-induced hTERT expression **(A)** GKN1 stable transfectants from passage 5 were transiently transfected with c-myc. GKN1 completely inhibited the binding activity of c-myc to a *hTERT* gene promoter in the ChIP assay. **(B)** Quantitative ChIP-qPCR assay of hTERT revealed that hTERT promoter occupancy increased in c-myc-transfected cells. However, it was strongly reduced in GKN1 stably expressed cells. (n = 3; error bars represent SEM; *p < 0.05 for a one-sided paired t-test). **(C and D)** Immunoprecipitated c-myc or TRF1 proteins were resolved by SDS-PAGE, transferred to PVDF membranes, and processed for immunoblotting with the indicated antibodies. The GKN1 protein in stable AGS^GKN1^ cells bound to c-myc and reduced its expression. In addition, the TRF1 protein bound to the c-myc protein in stable AGS^GKN1^ cells transiently transfected with *c-myc* and *TRF1*.

To determine the molecular mechanism of GKN1-induced down-regulation of hTERT, we examined the binding activity of c-myc to a *hTERT* promoter in AGS^GKN1^ and MKN1^GKN1^ cells from passage 5 after transient transfection with *c-myc*. Both Chromatin immunoprecipitation (ChIP) and immunoprecipitation assays were carried out, and the results are shown in Figure 5. In ChIP assay, binding activity of c-myc to a *hTERT* promoter was completely inhibited in GKN1 stable cells (Figure 5A). In addition, ectopic expression of c-myc markedly increased its binding to a *hTERT* gene promoter in AGS and MKN1 cells, whereas GKN1 completely suppressed c-myc binding in both cell lines (Figure 5B). GKN1 directly bound to the c-myc protein in the immunoprecipitation assay and down-regulated its expression (Figure 5C), as well as inhibited its binding activity to TRF1 (Figure 5D).

### Telomere length in non-cancerous gastric mucosa and cancer tissues

**Figure 6:**
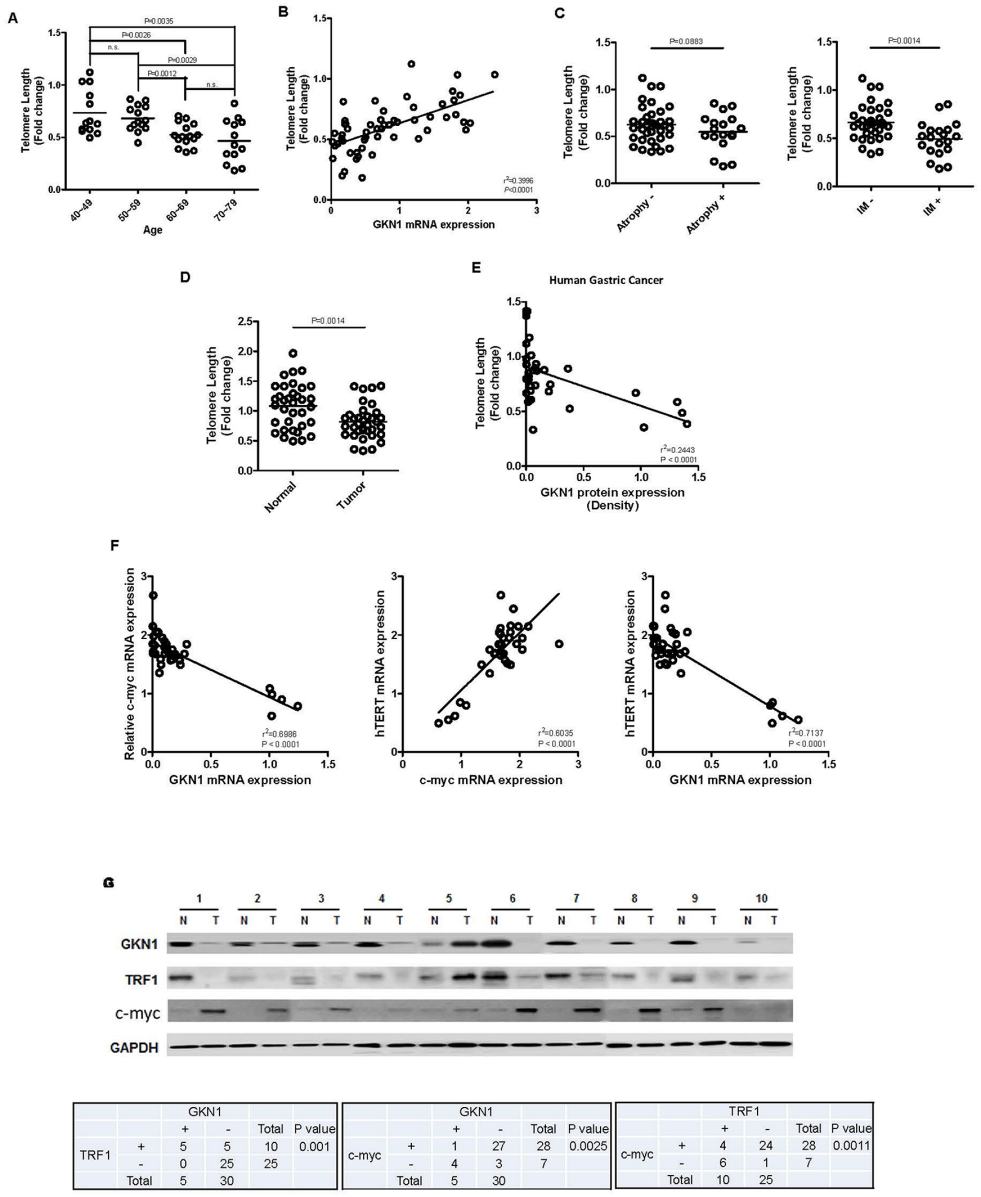
Correlation between GKN1 and telomere length in gastric mucosa and gastric cancer **(A)** Telomere length decreased steadily with aging in non-cancerous gastric mucosa tissues. The fold change in telomere length was calculated by subtracting the mean Ct value of 55 non-cancerous gastric mucosae from the Ct value of each sample. **(B)** Telomere length in 55 gastric mucosal tissues was closely associated with *GKN1* mRNA expression level. **(C)** Telomere length was significantly shorter in the gastric mucosal tissues with intestinal metaplasia (IM) than in cases without IM (P = 0.0014), whereas no association between telomere length and gastric mucosal atrophy was observed. **(D)** Telomeres shortened significantly in gastric cancer tissues, compared with those in non-cancerous gastric mucosae. **(E)** The GKN1 protein expression level in gastric cancer tissues was inversely correlated with telomere length. **(F)** An inverse association between *GKN1* mRNA and *c-myc* and *hTERT* mRNA transcript expression levels, and a positive correlation between *c-myc* mRNA and *hTERT* mRNA expression were detected in gastric cancer tissues. **(G)** A positive correlation was observed between GKN1 and TRF1 protein expression, and an inverse association was found between c-myc with GKN1 and TRF1 protein expression in gastric cancer tissues. N, non-neoplasic gastric mucosa; T, gastric cancer.

Telomeres steadily shorten with age, and their length was positively associated with GKN1 expression in 55 gastric mucosae, suggesting that GKN1 may be involved in the maintenance of telomere length in non-cancerous gastric mucosal cells (Figure 6A and 6B). In addition, shortening of telomeres was observed in gastric mucosa with intestinal metaplasia (Figure 6C). When we compared telomere length in 35 gastric cancer tissues with that of corresponding non-neoplastic gastric mucosae, we found that telomeres were significantly shorter in gastric cancers (Figure 6D) (P = 0.0014), and an inverse correlation was detected between telomere length and GKN1 protein expression (Figure 6E, P < 0.0001). Interestingly, *c-myc* mRNA expression was positively correlated with *hTERT* expression, whereas *GKN1* expression was inversely correlated with *c-myc* and *hTERT* mRNA expression in 35 gastric cancer tissues (Figure 6F). Furthermore, GKN1 protein expression was positively and inversely correlated with TRF1 and c-myc protein expression (Figure 6G).

## DISCUSSION

Telomeres play an important role in the maintenance of genomic stability and aberrant deregulation of telomeres has been well documented in gastric cancer [17]. Here, we provide molecular evidence for the involvement of GKN1 in the regulation of telomere length and telomerase activity through direct binding to c-myc, resulting in senescence and apoptosis in gastric cancer cells.

We and others have reported previously that GKN1 has a tumor suppressor function through inhibition of cell proliferation and induction of apoptosis in gastric cancer cells [13, 14, 18, 19]. Here, to investigate whether GKN1 plays a role in senescence and apoptosis of gastric cancer cells, we stably reconstituted GKN1 into AGS and MKN1 gastric cancer cells. The results show that AGS^GKN1^ and MKN1^GKN1^ stable cells grew at a much slower rate compared to that of mock-transfected cells (Figure 1A). Average telomere length and telomerase activity decreased steadily with advancing passages, and the PDLs were significantly lower in AGS and MKN1 cells transiently and stably transfected with GKN1 (Figure 1B-D). In addition, GKN1 shortened telomeres and diminished telomerase activity and hTERT expression in AGS and MKN1 cells (Figure 1E). This result was further substantiated by the observation that telomeres were shortened in gastric cancer tissues, compared to that in non-cancerous gastric mucosa, and that it was inversely correlated with GKN1 expression (Figure 6E). These results suggest that GKN1 plays an important role in telomere shortening in gastric cancer cells and appear to support the fact that the vast majority of human cancers have critically short telomeres but at the same time show reactivation of telomerase [20].

In human cells, both TRF1 and telomerase contribute to protecting and maintaining telomeric DNA. TRF1 overexpression results in a gradual shortening of telomeres, and a dominant negative allele of TRF1 elongates telomeres [21]. Direct interaction of TRF2 with Ku70 leads to the maintaining of telomeric-circle formation [22], which was important to prevent non-homologous end joining at the telomeres [23]. Telomerase, which adds TTAGGG sequences onto the 3′ ends of telomeric DNA and promotes genomic stability, is a critical determinant of telomere length [24]. To identify molecular mechanisms underlying GKN1-induced shortening of telomeres, we analyzed the expression levels of telomere-regulators, including TRF1, hTERT, and c-myc in AGS and MKN1 cells transiently and stably transfected with *GKN1*. Interestingly, marked up-regulation of the TRF1 protein and down-regulation of the hTERT protein were observed in *GKN1*-transfected AGS and MKN1 cells (Figure 2A). In stable cells, GKN1 similarly increased expression of TRF1 and reduced hTERT and c-myc protein expression in a time dependent manner, particularly at the chromatin level of gastric cancer cells (Figure 2B-E). Additionally, we found that TRF1 inhibited cell growth and telomerase activity, as well as shortened the telomeres in AGS and MKN1 cells (Supplementary Figure S1A-C)*.* Therefore, our data suggest that GKN1 may play a negative role in homeostasis of telomere length in gastric cancer cells by regulating the expression of telomere-related proteins.

As the critical shortness of telomeres and the inhibition of telomerase activity lead to apoptosis and senescence [24, 25], we investigated whether GKN1 could induce gastric cancer cells to undergo senescence and apoptosis by regulating telomere length. AGS^GKN1^ and MKN1^GKN1^ cells displayed obvious SA-β-gal staining at 24 and 48 hr after cell culture (Figure 3A and 3B). Cellular senescence decreased dramatically after 72 hr in GKN1-expressing cells, whereas the percentage of apoptotic cells increased significantly in a time-dependent manner (Figure 3B). Moreover, the cleaved form of caspase-3 was found in GKN1-reconstituted cells 48 hr after culture (Figure 3C). Critically short telomeres, associated with replication exhaustion, force cells to undergo either senescence or apoptosis [26]. Although the molecular mechanisms underlying cellular senescence followed by the activation of apoptotic program remain to be fully elucidated, we provide strong evidence for the acute effect of GKN1 on senescence in gastric cancer cells, leading to cell cycle arrest and eventually promoting apoptosis. In addition, our data suggest that the ability of GKN1 to induce apoptosis is an alternative way to eliminate senescent cells in gastric cancer.

Among several targets and regulators linked to the induction of senescence, p53 and p21 play a crucial role in the stimulation of cell cycle arrest and senescence, and inactivation of p21 is sufficient to bypass senescence [27, 28]. When the cell cycle is arrested, an inappropriate growth-promotion converts an arrest into senescence [29]. In addition, pharmacological inhibition of Skp2 leads to cellular senescence [30]. One study demonstrated that GKN1 induces senescence by activating the Ras/Raf/MEK/ERK pathway and its downstream p16/Rb and p21^waf^ effectors in gastric cancer cells [15]. Similarly, we found that expression of senescence-related proteins, including p53, p21, p27 and p16, was dramatically up-regulated, while Skp2 expression was down-regulated in GKN1 stable transfectants. Thus, these results suggest that GKN1 has the capacity to induce senescence by regulating the expression of senescence-related proteins as well as shortening telomeres in gastric cancer cells. Indeed, GKN1 did not affect p-ATM or p-ATR expression (Figure 3C), suggesting that GKN1-induced shortening of telomeres may not stimulate ATM- and ATR-dependent DNA damage response. This result agrees with the observation that the shelterin complex, including TRF1, serves to protect telomeres from being recognized as a DNA double strand break [4].

In stable GKN1 transfectants, treatment with CHX resulted in rapid degradation of TRF1, p53, and p27 proteins within 30 min (Figure 3D), whereas treatment with a proteasome inhibitor (MG-132) caused accumulation of these proteins (Figure 3E). Although the exact mechanism of the protein degradation remains unclear, it is possible that the GKN1-induced TRF1, p53 and p21 protein levels may have been regulated by post-translational degradation and transcriptional activation.

As c-myc is a transcriptional factor that interacts directly with the *hTERT* promoter and activates hTERT expression [31], we examined the effect of GKN1 on cell growth, telomere length, telomerase activity, and *hTERT* mRNA expression in mock controls and after transient transfection with *c-myc*. c-myc stimulated cell growth in AGS and MKN1 cells, whereas GKN1 completely inhibited c-myc-induced cell growth (Figure 4A). In addition, c-myc induced elongation of telomeres and increased telomerase activity in these cells (Figure 4B and 4C). However, when we transfected *c-myc* in both GKN1 stable cells*,* a significant decrease in telomere length and telomerase activity was observed (Figure 4B and 4C). Furthermore, GKN1 markedly down-regulated c-myc-induced *hTERT* mRNA expression (Figure 4D). The *myc* promoter is targeted by multiple signal transduction cascades, including the WNT, JAK/STAT, and NF-κB pathways, which are deregulated in cancer cells and contribute to enhanced myc expression [32, 33]. In our recent studies, GKN1 showed a negative correlation with the nuclear factor-kB [34] and β-catenin signaling pathways [35]. Although it is unclear how GKN1 controls expression and stability of the c-myc protein, these results suggest that GKN1 may inhibit cell growth by suppressing c-myc-induced telomere elongation in gastric cancer cells.

The fact that GKN1 regulates c-myc-induced expression of the *hTERT* gene prompted us to explore the impact of GKN1 on c-myc binding activity to the *hTERT* gene. Interestingly, GKN1 directly bound to the c-myc protein and down-regulated its expression, as well as completely suppressed its binding to a *hTERT* promoter (Figure 5A-C). Kim and Chen reported that c-myc blocks the inhibitory effect of TRF1 on telomere replication through a direct protein-protein interaction; thus, leading to an increased telomere length when it is overexpressed [36]. In the present study, GKN1 inhibited the interaction between the c-myc and TRF1 proteins (Figure 5D). Taken together, these findings suggest that GKN1 may affect the telomere length maintenance pathways by inhibiting not only c-myc-induced hTERT expression but also TRF1 inactivation in gastric cancer cells.

When we examined the association between GKN1 and telomere length in non-cancerous gastric mucosal tissues, telomeres became shorter with age, and shortening was closely associated with the presence of intestinal metaplasia (Figure 6A and 6C). In contrast to that of gastric cancer tissues, telomere length in the gastric mucosal tissues expressing GKN1 was longer (Figure 6B, 6D and 6E). Normal somatic cells do not usually activate telomerase to counter telomere shortening, and the gastrointestinal epithelium is characterized by a very high cellular turnover rate, which leads to renewal of the epithelium every 3–5 days [10, 37]. The results of our study present for the first time that GKN1 may play a role in telomere maintenance by inhibiting c-myc-induced telomerase activation in gastric epithelial cells and that loss of GKN1 expression in gastric cancer cells may activate telomerase. In addition, oxidative stress is an important factor associated with accelerated shortening of telomeres [38]. GKN1 inhibits reactive oxygen species (ROS) production by up-regulating antioxidant enzyme activities [35]. Thus, our data suggest that GKN1 may contribute to the physiological erosion of telomere length in gastric mucosal epithelial cells by regulating the activation of telomerase and aberrant telomere shortening by ROS. However, further studies are needed to identify the molecular regulatory mechanisms of GKN1-dependent telomere length in normal gastric epithelial cells.

In conclusion, we demonstrated an additional function of the GKN1 gastric tumor suppressor gene, which induces shortening of telomeres by acting as a potential c-myc inhibitor, leading to senescence and apoptosis in gastric cancer cells. Additional GKN1 functional and translational studies will provide a more comprehensive understanding of gastric epithelial homeostasis and gastric carcinogenesis. GKN1 may be a potential chemotherapeutic candidate for gastric cancer treatment, which will ultimately achieve the goal of gastric cancer prevention and remission.

## MATERIALS AND METHODS

### Cell culture and transfection of GKN1, c-myc, and TRF1

We generated stable transfectants of AGS and MKN1 cells, which stably expressed the GKN1 protein (AGS^GKN1^ and MKN1^GKN1^ cells). As shown in Figure 1A, Western blot analysis of AGS^GKN1^ and MKN1^GKN1^ cells demonstrated marked GKN1 protein expression. The effects of GKN1 and c-myc on cell growth, telomere length, telomerase activity, and hTERT expression were examined in stable AGS and MKN1 cells (AGS^Mock^, AGS^GKN1^, MKN1^Mock^, and MKN1^GKN1^). We also studied the impact of TRF1 on telomeres in transiently transfected AGS and MKN1 cells. The MTT [3-(4,5 dimethylthiazol-2-yl)-2,5-diphenyltetrazoliumbromide] assay was performed for cell viability analysis. Absorbance was measured at 540 nm using a spectrophotometer, and cell viability was expressed relative to that in the mock control. Cell culture and transfection of *GKN1*, *c-myc,* and *TRF1* are described in the Supplementary Materials and Methods.

### *In vitro* lifespan measured by cumulative population doubling level (PDL)

At each subcultivation, confluent AGS^GKN1^ and MKN1^GKN1^ cells were trypsinized and reseeded at a density of 1 × 10^5^ cells per dish. The cumulative PDL at each subcultivation was calculated from the cell count using the following equation [39]. PD = [log_10_(*N*_H_)-log_10_(*N*_1_)]/log_10_(2), where *N*_H_ is the number of harvested cells, and *N*_1_ is the number of seeded cells. An increase in population doubling was calculated and added to the previous PDL to yield cumulative PDL. The end of the replicative lifespan was defined by failure of the population to double after 13 passages in culture with 12 passages of consecutive refeeding in AGS and MKN1 cells stably expressing GKN1, as described previously [39].

### Measurement of telomere length and telomerase activity in gastric cancer tissues and cell lines

Telomere length was examined in AGS and MKN1 cell lines and 35 frozen advanced gastric cancers and corresponding non-cancerous gastric mucosal tissues by real-time quantitative polymerase chain reaction (qPCR). All patients underwent surgery without preoperative chemotherapy or radiotherapy. According to Lauren's classification, 23 cases were of the intestinal-type and 12 were of the diffuse-type gastric cancer. We obtained primary cancer tissues and corresponding surrounding gastric mucosal tissues. Tissue slices were stained with hematoxylin and eosin and examined by experienced pathologists. To investigate whether telomere length is associated with the degree of gastritis, an additional 55 non-neoplastic gastric mucosal tissues were included. Histological assessment was performed independently by two pathologists, as described previously [40]. After quantifying the genomic DNAs extracted from each sample, real-time SYBR Green qPCR was performed on a Stratagene Mx 3000P qPCR system (Stratagene, La Jolla, CA, USA). The specific primers for detecting telomere length in gastric tissues and cell lines were designed according to data reported previously [41]. All samples were subjected to PCR amplification with specific oligonucleotide primers for the constitutively expressed single gene copy number (36B4) and normalized. Briefly, the ratio of telomere repeat copy number to single-copy gene number (T/S ratio) was determined using the comparative Ct method. Sample T/S ratios were then divided with the T/S ratio of a reference DNA included in each plate, generating relative telomere length values. Each sample was loaded in triplicate, and all PCR-plates included a standard curve for PCR efficiency calculations. Primer Sequences are described in Supplementary Table S1. Written informed consent was obtained from all participants in accordance with the Declaration of Helsinki. This study was approved by the Institutional Review Board of The Catholic University of Korea, College of Medicine (CUMC09U089).

Telomerase activity was measured in CHAPS extracts (250 ng) from the AGS and MKN1 cell lines, using a quantitative telomerase detection kit (QTD kit, Allied Biotech Inc., Benicia, CA, USA). QTD real-time PCR assays were performed on the Stratagene Mx 3000P QPCR system according to the manufacturer's protocol. The kit also included a telomerase substrate oligonucleotide (TSR) control template, providing a standard curve and a positive control. Telomerase activity was evaluated using the relative quantities according to an internal calibrator using the 2-^ΔΔ^CT method [42].

We also analyzed telomere length and telomerase activity at 2, 5, 8, 11, and 14 passages and at 1, 2, 3, and 4 days in stable AGS^Mock^, MKN1^Mock^, AGS^GKN1^, and MKN1^GKN1^ cells from passage 1 and in AGS and MKN1 cells transiently transfected with *c-myc* or *TRF1*.

### Expression of the proteins regulating telomere length

To determine whether GKN1 is involved in regulating telomere length, the expression of telomere-related proteins, including TRF1, hTERT, c-myc, p53, p21, p16, p27, p-ATM, p-ATR, Skp2, and caspase-3 was examined in AGS^Mock^, MKN1^Mock^, AGS^GKN1^ and MKN1^GKN1^ cells at 24, 48, 72 and 96 hr*.* The antibodies used are listed in Supplementary Table S2.

Total protein extracts for chromatin fractionation were isolated in RIPA buffer [1 × PBS, 1% (v/v) NP-40, 0.5% (w/v) sodium deoxychelate, and 0.1% (w/v) SDS] supplemented with 1 mM Pefabloc and 1 ng/μl aprotinin/leupeptin. The soluble and insoluble nuclear extracts were prepared as described previously [43]. Briefly, cells were washed twice with PBS before resuspending in buffer A (10 mM HEPES, pH 7.9, 10 mM KCl, 1.5 mM MgCl2, 0.34 M sucrose, 10% (v/v) glycerol, 1 mM DTT, 0.1% (v/v) Triton X-100, supplemented with protease inhibitors: 1 mM Pefabloc, 1 ng/μl aprotinin/leupeptin and 10 mM β-glycerophosphate) and incubated in ice for 5 min. Nuclei were isolated by centrifugation at 1,300 × g for 5 min at 4°C, washed once with buffer A (depleted of Triton X-100) and then lysed in buffer B (3 mM EDTA, 0.2 mM EGTA, 1 mM DTT plus supplements as in buffer A). Soluble and insoluble (chromatin) fractions were separated via centrifugation at 1,700 × g for 4 min at 4°C. Chromatin samples were subsequently resuspended in buffer B. The fractionated cell lysates were separated on a 10% polyacrylamide gel and transferred onto a Hybond PVDF membrane (Amersham Pharmacia Biotech, Piscataway, NJ, USA). After blocking, the membrane was probed with antibodies against telomere-related proteins. Protein bands were detected using enhanced chemiluminescence reagents (Amersham Pharmacia Biotech).

### Measurement of senescence and apoptosis

All senescence and apoptosis experiments were performed in cells from passage 5. Detail describing the procedures for the senescence and apoptosis analysis are described in Supplementary Materials and Methods.

### Chromatin immunoprecipitation (ChIP)

ChIP assays were performed using the Thermo Scientific Pierce Agarose ChIP kit (Thermo Scientific Pierce, Rockford, IL, USA). AGS^Mock^ and AGS^GKN1^ cells from passage 5 were cultured in a 10-cm dish and incubated in RPMI-1640 medium with 10% fetal bovine serum for 4 days after transfection with mock or c-myc. The cells were fixed with 1% formaldehyde in PBS for 10 min, washed twice with ice-cold PBS and resuspended in lysis buffer. Nuclei were recovered by centrifugation and MNase digestion was carried out at 37°C for 15 min. Nuclei were lysed and the extracts were immunoprecipitated with 4 μg of antibody against c-myc at 4°C overnight. Normal rabbit IgG was used as the negative control. Immune complexes were collected with Protein A agarose and washed, then cross linking between proteins and DNA was reversed by incubation at 65°C for 40 min. Protein-bound DNA was recovered using affinity chromatography purification columns according to the manufacturer's protocol (Thermo Scientific), and 5 μl of lysed nuclei were also purified under the same procedure and used as input. DNA amplification was performed by PCR using primers for the *hTERT* promoter described in Supplementary Table S1. Amplification products were separated on a 2% agarose gel.

*hTERT* promoter and enhancer expression were quantified by SYBR Green-based real-time qPCR. All samples were subjected to PCR amplification with oligonucleotide primers specific for the constitutively expressed input of *hTERT* promoter DNA and normalized.

### Protein stability assay

To measure stability of the telomere-related proteins, AGS^Mock^, MKN1^Mock^, AGS^GKN1^, and MKN1^GKN1^ cells from passage 5 were incubated for 22 hrs and treated with 50 μg/ml CHX (Sigma, St. Louis, MO, USA) and 10 μg/ml of the proteasome inhibitor MG-132 (Sigma). At 0.5, 1, 2, 4, and 6 hr after treatment, the total cell lysates were isolated to detect intracellular protein abundance via Western blot.

### Measurement of *GKN1* mRNA expression in gastric cancer tissues

*GKN1* mRNA expression was examined in non-cancerous gastric mucosa and cancer tissues by real time reverse transcription PCR, as described previously [14]. After cDNA synthesis, 50 ng cDNA was amplified using Fullvelocity SYBR Green QPCR Master Mix (Stratagene) and 20 pmol/μl each of the forward and reverse primers on the Stratagene Mx 3000P QPCR system.

GKN1, TRF1, and c-myc protein expression was examined in 35 advanced gastric cancers by Western blot analysis, as described previously [13]. We determined the GKN1 expression levels in gastric cancer tissues by densitometer after Western blot.

### Statistical analysis

Student's *t*-test was used to analyze the effect of GKN1 on cell growth, telomere length, and telomerase activity. Data are expressed as means ±S.D. from at least three independent experiments. The association between *GKN1* mRNA expression level and telomere length in gastric tissues was tested using the chi-Square or Spearman's correlation test. A *P*-value ≤ 0.05 was considered to be significant.

## SUPPLEMENTARY MATERIALS, TABLES AND FIGURE


