# Supplementary material for: Gastrokine 1 induces senescence and apoptosis through regulating telomere length in gastric cancer
Source: Oncotarget. 2014 Dec 10;5(22):11695–708. doi: 10.18632/oncotarget.2586 (PMC4294346; doi:10.18632/oncotarget.2586)
Supplement: Supplementary file 1 [file oncotarget-05-11695-s001.pdf]

SUPPLEMENTARY MATERIALS, TABLES AND FIGURE

Cell culture and transfection of GKN1, c-myc and TRF1

AGS and MKN1 gastric cancer cells were obtained from the Korean Cell Line Bank (KCLB, Seoul, Korea). These cell lines were cultured at 37°C in 5% CO_2 in RPMI-1640 medium (Lonza, Basel, Switzerland) with 10% heat-inactivated fetal bovine serum. Cells were authenticated by short tandem repeat profiling by KCLB, and were used within 12 months after receipt or resuscitation. For transient transfection, *GKN1, c-myc,* and *TRF1* cDNAs were cloned into the pcDNA3.1 expression vector (Invitrogen, Carlsbad, CA, USA). AGS and MKN1 cells were transiently transfected in 60 mm-diameter dishes with the expression plasmids (2 µg total DNA) using Lipofectamine Plus transfection reagent (Invitrogen) according to the manufacturer's recommendations. Transfection rate was monitored by fluorescence microscopy, and the transfection efficiency of more than 80% was achieved.

Generation of stable cell lines expressing GKN1

To isolate stable cell lines expressing GKN1, AGS and MKN1 cells were seeded into 100-mm dishes. When the cells reached 80% confluence, the human GKN1-expressing vector was transfected into AGS and MKN1 cells using Lipofectamine 2000 (Invitrogen). After 24 h, the medium was changed, and G418 (Wako, Osaka, Japan) was added to the culture medium at a final concentration of 1 mg/mL. Thereafter, the cells were cultured in the presence of G418 for 4 weeks. Medium was exchanged every 2 days. Some cells were isolated from the colonies formed in the culture and were seeded into other dishes to establish a stable cell line expressing GKN1. The expression of GKN1 in each cell line was determined by immunoblot analysis.

Measurement of senescence and apoptosis

For senescence analysis, all the experiments were performed in cells from passage 5. The cells (2×10^6) were cultured in a 10-cm dish and incubated for 4 days in RPMI-1640 medium with 10% FBS. When the cells reached approximately 80% confluence, serial passage was performed by trypsinization. Stable AGS^{Mock}, $MKN1^{Mock}$, AGS^{GKN1}, and $MKN1^{GKN1}$ cells were fixed and incubated with freshly prepared senescence-associated β-galactosidase staining solution at 37°C overnight. The number of blue-stained cells was counted in at least 10 fields at 20X magnification and expressed as the percentage of the senescent cells.

For apoptosis analysis, cells (2×10^6) from passage 5 were cultured in a 10-cm dish and incubated for 4 days in RPMI-1640 medium with 10% FBS. For annexin V staning, AGS^{Mock}, $MKN1^{Mock}$, AGS^{GKN1}, and $MKN1^{GKN1}$ cells were washed twice with 1 X binding buffer and incubated with freshly prepared 1 X binding buffer, which included annexin V, fluorescein isothiocyanate (FITC) and 4′,6-diamidino-2-phenylindole (DAPI) at room temperature for at least 30 min in the dark. After staining, the observation was done under a fluorescence microscope (Carl Zeiss Co., Ltd., Germany)

Supplementary Table S1. Real-time RT-PCR primer sequences

Gene	Primer sequences
hTERT	F: 5′- ATG CGA CAG TTC GTG GCT CA-3′<br>R: 5′- ATC CCC TGG CAC TGG ACG TA-3′
c-Myc	F: 5′- CCT ACC CTC TCA ACG ACA GC -3′<br>R: 5′- CTC TGA CCT TTT GCC AGG AG -3′
Telomere standard	5′- TTA GGG TTA GGG TTA GGG TTA GGG TTA GGG TTA GGG TTA GGG TTA GGG TTA GGG TTA GGG TTA GGG TTA GGG TTA GGG TTA GGG -3′
36B4 standard	5′- CAG CAA GTG GGA AGG TGT AAT CCG TCT CCA CAG ACA AGG CCA GGA CTC GTT TGT ACC CGT TGA TGA TAG AAT GGG -3′
Telomere	F: 5′- CGG TTT GTT TGG GTT TGG GTT TGG GTT TGG GTT TGG GTT -3′<br>R: 5′- GGC TTG CCT TAC CCT TAC CCT TAC CCT TAC CCT TAC CCT -3′
36B4	F: 5′- CAG CAA GTG GGA AGG TGT AAT CC -3′<br>R: 5′- CCC ATT CTA TCA TCA ACG GGT ACA A -3′
GKN1	F: 5′- CAA AGT CGA TGA CCT GAG CA -3′<br>R: 5′- CTT GCC TCT TGC ATC TCC TC -3′
GAPDH	F: 5′- AAA TCA AGT GGG GCG ATG CTG -3′<br>R: 5′- GCA GAG ATG ATG ACC CTT TTG -3′
hTERT promoter for CHIP	F: 5′- AGT GGA TTC GCG GGC ACA GA -3′<br>R: 5′- TTC CCA CGT GCG CAG CAG GA -3′

Supplementary Table S2. Antibody list for western blot analysis

Gene name	Company
GKN1	Sigma
c-Myc	Cell signaling
TRF1	Santa cruz
hTERT	Abcam
p27	Cell signaling
p53	Cell signaling
p21	Cell signaling
p16	Santa cruz
p-ATM	Cell signaling
p-ATR	Cell signaling
Skp2	Santa cruz
Caspase 3	Cell signaling
GAPDH	Santa cruz
α-tubulin	Santa cruz
Histone H4	Cell signaling

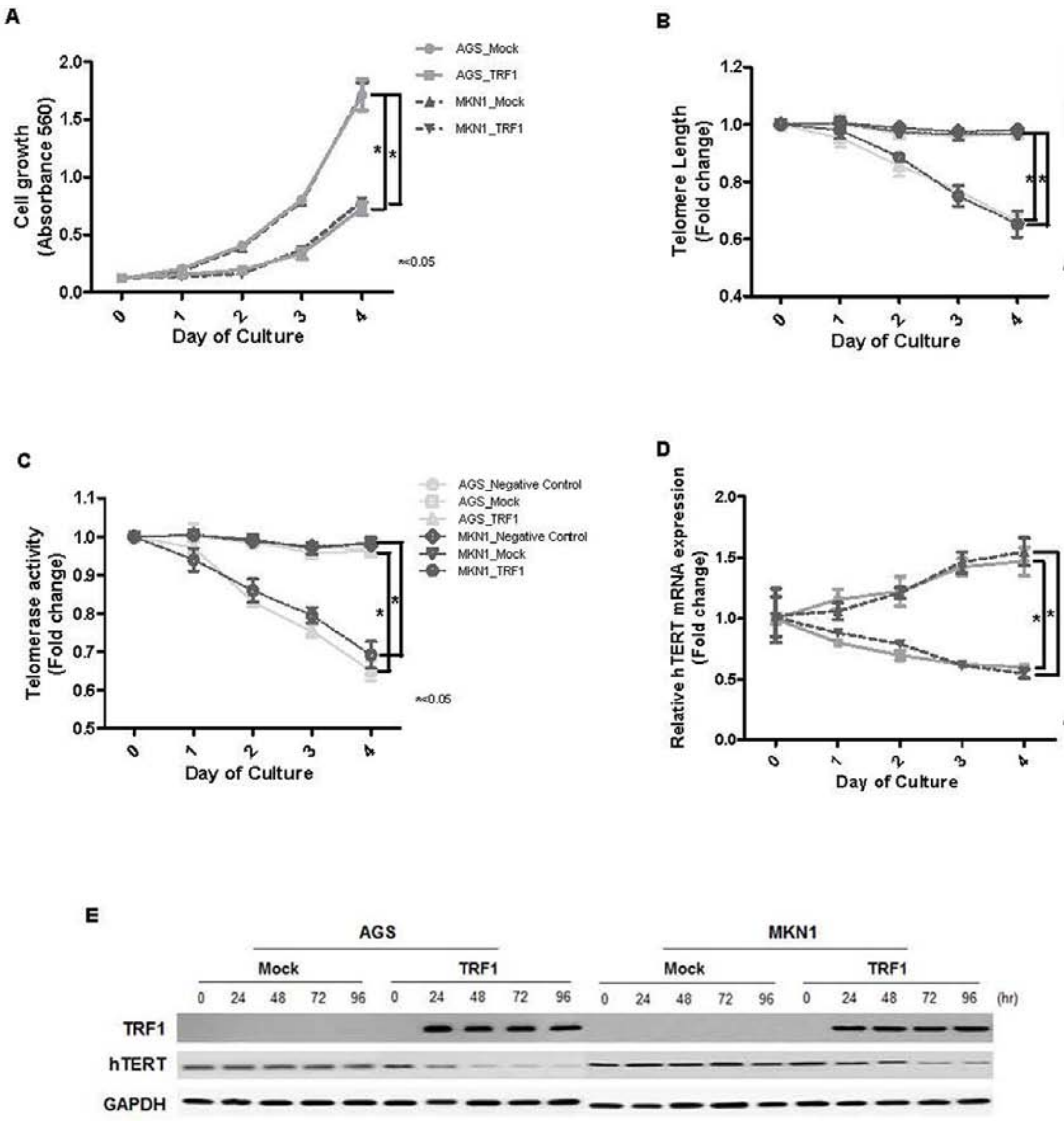

Supplementary Figure S1: TRF1 inhibits cell viability and decreases telomere length and telomerase activity (A) In MTT assay, ectopic expression of TRF1 significantly inhibited cell viability of AGS and MKN1 cells. **(B, C & D)** There was a steady decline in the telomere length (B), telomerase activity (C), and *hTERT* mRNA transcript (D) in AGS and MKN1 cells transfected with *TRF1*, compared with those in non-transfected negative control and mock-transfected cells. **(E)** Ectopic expression of TRF1 markedly reduced the hTERT protein expression in both cells.
